# RNA Viruses: ROS-Mediated Cell Death

**DOI:** 10.1155/2014/467452

**Published:** 2014-05-08

**Authors:** Mohammad Latif Reshi, Yi-Che Su, Jiann-Ruey Hong

**Affiliations:** ^1^Laboratory of Molecular Virology and Biotechnology, Institute of Biotechnology, National Cheng Kung University, Tainan 701, Taiwan; ^2^Department of Life Sciences, College of Bioscience and Biotechnology, National Cheng Kung University, Tainan 701, Taiwan

## Abstract

Reactive oxygen species (ROS) are well known for being both beneficial and deleterious. The main thrust of this review is to investigate the role of ROS in ribonucleic acid (RNA) virus pathogenesis. Much evidences has accumulated over the past decade, suggesting that patients infected with RNA viruses are under chronic oxidative stress. Changes to the body's antioxidant defense system, in relation to SOD, ascorbic acid, selenium, carotenoids, and glutathione, have been reported in various tissues of RNA-virus infected patients. This review focuses on RNA viruses and retroviruses, giving particular attention to the human influenza virus, Hepatitis c virus (HCV), human immunodeficiency virus (HIV), and the aquatic Betanodavirus. Oxidative stress via RNA virus infections can contribute to several aspects of viral disease pathogenesis including apoptosis, loss of immune function, viral replication, inflammatory response, and loss of body weight. We focus on how ROS production is correlated with host cell death. Moreover, ROS may play an important role as a signal molecule in the regulation of viral replication and organelle function, potentially providing new insights in the prevention and treatment of RNA viruses and retrovirus infections.

## 1. Introduction

Cellular metabolisms produce different varieties of reactive oxygen species (ROS) as byproducts. These ROS play an important role in cell signaling and regulate hormone action, growth factors, cytokines, transcription, apoptosis, ion transport, immunomodulation, and neuromodulation [1, 2]. They lend fundamental aid to the normal functioning of the body's immune system and proliferate T-cells that provide immunological defense (adaptive immunity) [3, 4]. However, when the same ROS are produced by activated neutrophiles, macrophages destroy microbes/viruses and neighboring cells via oxidative bursts [5]. Any imbalance in the production of ROS and the body's inability to detoxify these ROS is referred to as oxidative stress [6]. SOD and catalases are the major defense against the ROS produced in cells [7, 8]. The research has shown that children suffering from hepatitis B or C exhibit increased levels of lipid peroxidation, which indicates weak antioxidant defense due to low catalase and SOD activity [9]. Earlier and recent studies have suggested that ROS induces apoptosis [10–12], and that the agents that cause apoptosis are either oxidants or generate the ROS. This hypothesis was later shown to be correct when researchers demonstrated the role of proto-oncogene BCL-2 in preventing apoptosis in an antioxidant way [13].

Peterhan and his coworkers were the first to demonstrate that a virus could generate ROS from phagocytes [14]. Later research showed that many retroviruses, DNA viruses and RNA viruses can cause cell death by generating oxidative stress in infected cells [15–17]. In 1994, the scientific community held its first conference to discuss the possible interaction between viral infection and ROS in detail [18]. RNA viruses generally use RNA as their genetic material, and those that use DNA intermediates in their replication cycle are known as retroviruses. These viruses posses the highest mutation rates among every living creature [19–22]. Therefore, it is not always easy to develop successful and effective vaccines and drugs against these viruses. Oxidative stress always plays a dominant pathogenic role in HIV and hepatitis infections. AIDS is the end phase of HIV infection. This pandemic is caused by the HIV-1 and HIV-2 groups of cytopathic viruses, wherein the levels of GSH, cystine, vitamin C and SOD are decreased and the MD and HNE levels are elevated in patients infected with HIV-1 [23–25]. A decrease in antioxidants indicates the weakening of the immune system, as immune cells require more antioxidants to maintain their function and integrity. The CD4+ T-helper cells that form important components of the immune system are the main targets of the HIV virus. The virus production decreases to begin with, as about 5% of the T-cells are destroyed and replaced each day via the apoptotic process. This in turn leads to decreases in zinc and vitamin E (antioxidants). The decrease in zinc results in the inhibition of intracellular virus replication [26], and the selenium decrease indicates the progression of HIV toward AIDS. After suffering the primary illness, patients do not show any symptoms for up to more than 10 years, during which the virus load falls but the virus does not stop replicating. This in turn leads to a higher decrease in the CD4+ T-cell count, which ultimately leads to AIDS and the terminal stage of the infection. HIV-2 infection is slow compared with HIV-1 infection. In hepatitis patients, HIV preferentially infects CD4+ T-lymphocytes and macrophages. The hepatitis C virus belongs to the flaviviridae family of RNA with a positive strand RNA genome [27] at a size of 9,400 bp [28]. About 150 million people are infected, and have a higher chance of developing liver cancer/cirrhosis [29]. The World Health Organization reported that 80% of patients with acute hepatitis C progress toward chronic hepatitis, with 2% developing liver cirrhosis and 1–5% developing hepatitis C carcinoma [30, 31]. Researchers first exhibited the occurrence of oxidative stress during chronic hepatitis C in 1990 [32]. This OS is associated with hepatic damage, a decrease in GHS, an increase in serum malondialdrhyde (MDA), 4-hydroxynonenal (HNE) and caspase activity, and decreases in plasma and hepatic zinc concentrations [33–35]. Zinc therapy increases the functioning of surviving liver tissue [35]. However, zinc and selenium deficiencies affect DNA repair and the immune system, increasing the chances of chronicity and malignancy [36]. HCV replication takes place in hepatocytes, which potentially attack and propagate in immune system cells. The infection of different influenza viruses presents different clinical scenarios [37]. The influenza A virus is highly active, causing infection of the upper and lower respiratory tract. It can be divided into 16 different HA and NA combinations, with three HA (HA1, HA2 and HA3) and NA1 and NA2 being prevalent in humans [38]. Studies conducted in 19 different countries have shown that HINA and H3N2 are the most dominant influenza A viruses [39]. Influenza viruses and parmoviruses have been shown to activate monocytes and polymorphonuclar monocytes to generate ROS* in vitro* [15]. Activated phagocytes release not only ROS but also cytokine and TNF. The pro-antioxidant effect of TNF may be relevant to influenza virus infection because children with Rey's syndrome [40] exhibit increased levels of pro-oxidants and lipid peroxides. OS ultimately leads to a decrease in antioxidant levels and indicates a decrease in the functioning of the immune system. Immune system cells generally require a higher concentration of antioxidants than other cells to maintain the system's rodex balance and preserve its integrity and function.

## 2. Apoptosis

Apoptosis, the process of programmed cell death, involves a sequence of events that lead to various morphological changes in a cell including cell shrinkage, changes to the cell membrane such as the loss of membrane asymmetry and attachment, nuclear fragmentation, chromatin condensation, and genomic DNA fragmentation. Apoptosis has a key role in the pathogenesis of many diseases including cancer, inflammation and neurodegenerative diseases. Different modes of cell death are defined by morphological criteria. The term “apoptosis,” which was coined by kerr et al. [41], makes no clear reference to a precise biochemical mechanism, and is rather used to explain a special mode of cell death that is characterized by the rounding-up of cells, reduction in cell volume (pyknosis), condensation of chromatin, fragmentation of the nucleus (karyorrhexis) and maintenance of the intact plasma membrane until the very late stages of the process [42]. The process of programmed cell death is controlled by different ranges of cell signaling pathways originating from either the external surroundings of a cell (extrinsic inducers) or from within the cell itself (intrinsic inducers). Extrinsic inducers include heat, radiations, toxins [43], nitric oxide [44] and hormones. They must either pass into the cell or interact with the specific receptors present on the cell membrane to initiate the specific signal transduction pathway inside. This action leads the cell to undergo apoptosis. Thus, any external or internal disturbance in the signal transduction pathways within a cell, such as heat, radiation, viral infection, lack of nutrients or an increase in calcium concentration [45], leads the cell to undergo continuous proliferation or necrosis. Different kinds of cellular components such as polyADP ribose polymerase may also help regulate apoptosis [46]. Thus, any disturbance in the regulation of the apoptotic process either inside or outside a cell can lead to excessive apoptosis causing hypotrophy, such as in the form of ischemic damage, or result in an unchecked cell proliferation such as cancer. However, more than 139,000 research articles have been published in relation to the molecules involved in the activation of intrinsic and extrinsic apoptotic pathways such as BCL-2, TNF, NF-*κ*B and P53 [43]. Genetic studies related to the nematode* Caenorhabditis elegans* have shown that the molecules Ced-3 and Ced-4 (“Ced” for cell death abnormal) are important for the apoptosis of 131 cells during worm development [47]. These two molecules have been found to be the same as caspases. Caspases are the 30–60 KDa proenzymes inside cells. They are made continuously and activated by proteolytic processing either auto catalytically or in a cascade by enzymes with similar characteristics [48, 49]. This indicates that the apoptosis process is highly conserved. However, in the case of cell injury where an injured cell swells and bursts, the cell leaks its contents and attracts different immune cells such as lymphocytes by engaging in an unwanted inflammatory response [50].

## 3. Necrotic Cell Death

The term “necrosis” is currently used to describe accidental cell death, which mostly occurs due to cell injury resulting in the early death of cells in the affected area [51]. Therefore, necrosis is always detrimental and can even prove fatal. In the case of cell injury where the injured cell swells and bursts, the cells leaks its contents out and attracts different immune cells such as lymphocytes by engaging in an unwanted inflammatory response [50]. Nearby phagocytes are prevented from engulfing the dead cells [52], which in turn results in the formation of dead tissue. There is often no explanation for how a necrotic cell death occurs [53]. Therefore, necrosis lacks some of the features of apoptosis and autophagy. In addition, the clearance of necrotic cells operates differently from that of apoptotic cells [54].

## 4. Apoptosis in Virus Infected Cells

The perforin/granzyme pathway is often examined and highly involved in virus infection studies, which have found that CTLs and NK cells can eradicate virus-infected target cells via the apoptosis process [55]. Another important apoptosis-related host defense mechanism involves CTL, and recognizes and kills target cells by sending signals through cell surface death receptors [56]. Death receptors are a special type of receptor belonging to the tumor necrosis family (TNF). They contain specific homologous amino acid sequences in their cytoplasmic tails, referred to as the death domain (DD). The most well-studied death receptors include CD95/FAS and TNFRI, although additional death receptors such as DR3, 4 and 5 have been examined [56, 57]. In addition to killing virus-infected cells, death receptors aid in killing mature T-cells at the end of immune responses.

**Figure 1:**
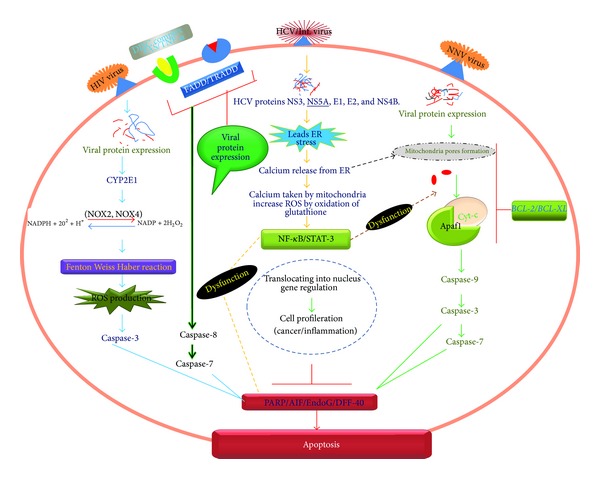
Schematic diagram of apoptotic cascade and the sites of action of general and specific caspases. The figure also illustrates how ROS produced during viral infection can affect apoptotic cascades. Abbreviations used in figure: FAS/TNF-*α*: tumor necrosis factor alpha (death receptors); FADD: Fas associated death domain; TRADD: tumor necrosis factor receptor associated death domain; Cas: caspase; AIF: apoptosis-inducing factor; PARP: poly (ADP) ribose polymerase; EndoG: endonuclease G; DFF-40: DNA fragmentation factor 40 KDa; tBID: truncated BID; DISC: death-inducing signaling complex.

In the case of FAS-mediated apoptosis, the interaction between the receptor and legend needs the help of adaptor proteins to signal the target cell to undergo the apoptosis process (Figure 1). The interaction between the two cells results in a clustering of intracellular DDs on the target cell. However, to be effective, this in turn activates the FADD adaptor protein within the target cell. In addition to interacting with the receptor [58], the FADD also contains a caspase-recruiting domain (CARD) that is responsible for activating the caspases (caspase-8). Upon oligomerization, the caspase-8 activates itself and the cascade of caspases (caspase-3) to begin the apoptosis process [58].

TNFR1- and DR3-type death receptors require FADD in addition to the TNFR-associated DD (TRADD) adaptor protein. TNFR1- and DR3-mediated cell deaths rarely occur unless and until protein synthesis is inhibited [59]. When the TNF-*α* pathway is activated, it can also suppress the apoptosis process inside the cell using different signaling pathways, which in turn stimulates the expression of antiapoptotic genes such as IAP, BCL-2, and BCL-XL and TNFR-associated factors that do not allow the activation of caspase family proteins [59–61]. The NF-*κ*B signaling pathway has proved to be important in cell death studies. Although a P53-mediated cell death requires the activation of the NF-*κ*B signaling pathway, the activation of NF-*κ*B by P53 is quite different because it requires MEKi (MEK inhibitor) and pp90rsk [62]. Recent research carried out on the Epstein Bar virus has shown that NF-*κ*B plays an important role in virus-mediated cell death compared with other signaling pathways. The activity of NF-*κ*B can be regulated by a number of viruses such as EBV through latent membrane protein-1 (LMP-1), which in turn enhance cell survival upon virus infection [63–65].

## 5. ROS Friend and Foe of Cells

**Figure 2:**
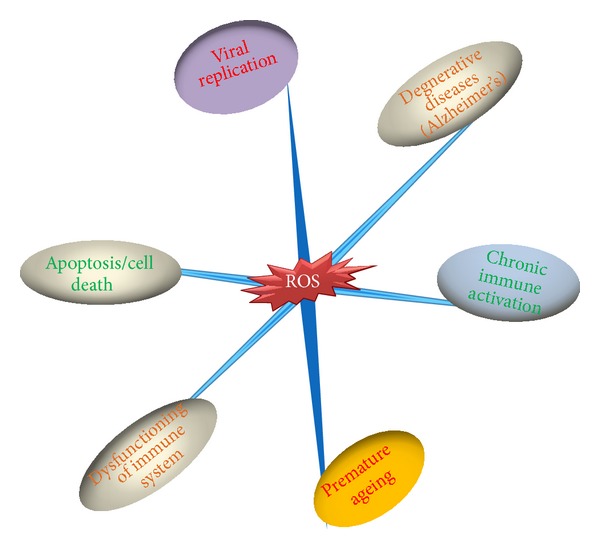
Effects of reactive oxygen species on different areas.

There are many different types of free radicals, but those of the greatest concern in biological systems are derived from oxygen. Excessive ROS production in a cell can lead to the oxidation of macromolecules and has been found to be responsible for causing mtDNA mutations, aging, and cell death. Any imbalance in the production of ROS is often referred to as oxidative stress [66, 67]. The effect of ROS on the cellular functions inside a cell depends on the amount of ROS and how much time the cell has been exposed to ROS (Figure 2). It has been confirmed multiple times that ROS act as both friend and foe to a cell. Oxidative stress has been found to play a role in various pathological conditions such as cancer, diabetes, and neurological disorders [68–71]. ROS include both the free radicals and nonradicals produced during various metabolic processes, mostly in the electron transport chains in mitochondria, peroxisomes, and ER stress and in nuclear and plasma membranes in aerobic cell metabolism. ROS are typically also produced during normal metabolic processes inside the cells. They are neutralized by the antioxidant defense system, including enzymes such as SOD, free-radical scavengers, and metal chelates. However, nutrition makes the most significant contribution to the body's antioxidant defense system. It is widely believed that diet-derived antioxidants play a role in the prevention of human diseases. These antioxidants work in a coordinated manner, where a deficiency in one may affect the efficiency of another. Semba and Tang reported that the low plasma or serum levels of vitamins A, E, B6, B12, and C; carotenoids; selenium; and zinc are common in many HIV-infected populations and may contribute to the pathogenesis of the HIV infection via increased oxidative stress and compromised immunity [72, 73]. Another research team showed that a deficiency of vitamin E or selenium allows the conversion of the normal benigh coxsackievirus virus B3 to virulence via a change in the nucleotide sequence in the genome of the benign virus and causes heart damage [74]. However, Evans and Halliwell showed that iron deficiency may serve as protection for malaria or Yersinia infections and that iron overload may make patients susceptible to infection by supplying enough iron for multiplying bacteria [75]. Therefore, the oxidative stress generated by micronutrient deficiencies becomes significant when disease or infection occurs. Any imbalance in the body's antioxidant defense system results in oxidative stress leading to cell damage. ROS and free radicals are generated in various human infectious diseases caused mostly by viruses in addition to bacteria and other parasites [71]. After entering a cell, a virus disturbs the cell's normal functioning by using the cell's machinery to replicate itself. This in turn leads to an imbalance in the cell's ROS system. Oxidative stress has been found to enhance viral replication in different viral infections [76–78].

ROS are usually generated during allergic and nonallergic inflammation in the body's inflammatory cells [79, 80]. Because they can act on the proteins and lipids in addition to other cell organelles, ROS can be considered part of the defense system against viral/bacterial infections. The ROS produced must be specific and also produced in limited amounts, as they can destroy the cell for which they are generated in addition to the cell's neighboring surroundings during highly inflammatory reactions [81, 82]. However, the ROS produced during the normal metabolic process vanish in the body's antioxidant pools such as catalases and SOD. In the case of bacterial and viral infections, ROS are produced by phagocytes to generate the respiratory burst [83–85] resulting from the NADPH oxidase activity. In addition, ROS can act as chemical messengers. Research has shown that ROS take part in both signaling pathways [86–88] and transcriptional activation [86–88].

### 5.1. Mitochondria: Source and Target of ROS

Although mitochondria are known as the powerhouses of a cell, they are the most suitable targets of the ROS produced inside a cell. These ROS mostly target the mtDNA, which encodes 13 polypeptides, 2 ribosomal RNA, and 22 tRNA [89]. All of these byproducts of mtDNA are essential components in electron transport chains for the generation of ATP via the oxidative phosphorylation process [90]. ATP generation requires proteins from both the nuclear genome and mitochondria. Therefore, the oxidative production of the ATP required for cellular function also generates ROS that can damage the mtDNA, membrane lipid permeability, release of cytochrome C into the cytosol, and activation of the key effector protease caspase-3 via proteolytic cleavage that ultimately results in the mitochondrial-mediated apoptosis pathway [91, 92]. Thus, any injury to mitochondria DNA can result in serious cell damage. The mtDNA is more suitable for ROS due to its lack of protective histones and its proximity to the electron transport chain, which is the main center of ATP production in mitochondria. Therefore, mitochondria are the major source of ROS production inside a cell, and Mother Nature has provided them with their own antioxidant defense system, the most important component of which is the glutathione GSH (reduced glutathione). Although there is no proof that glutathione biosynthesis occurs inside mitochondria, these organelles have their own distinct glutathione polls [93].

In the case of mitochondrial dysfunction, when released into cytoplasm, cytochrome C interacts with the apoptotic release factor (Apaf1) to initiate apoptosis (the mitochondrial-mediated apoptosis pathway). The proapoptotic gene Bax from the BCL family can cause mitochondria to release cytochrome C directly [94]. Along with other members of the BCL-2 family, Bax has the ability to create ion channels on the outer membrane of mitochondria, through which cytochrome C is released easily into the cytoplasm. Although how ROS act on mitochondria to release cytochrome C remains unknown, the ROS could cause MMP loss [93, 94], which can allow pore formation and the release of cytochrome C into cytoplasm, activating the cell death mechanism. However, it is unclear how ROS are initially released from mitochondria into the cytoplasm.

### 5.2. ROS in Endoplasmic Reticulum

Endoplasmic reticulum is mainly responsible for protein folding and assembly. It also acts as a primary storage house of calcium, which is required for the proper folding of proteins [95]. Any change in the normal function of endoplasmic reticulum results in the accumulation of misfolded and unfolded proteins, and changes in calcium homeostasis cause endoplasmic reticulum stress that finally leads to apoptosis [96]. Researchers currently believe that the oxidation of proteins in endoplasmic reticulum, which is associated with protein folding, is responsible for the generation of ROS that cause oxidative stress. This oxidative stress results in the leakage of calcium from endoplasmic reticulum lumen into cytoplasm [97–100]. Therefore rising Ca^2+^ concentration in the cytoplasm causes Ca^2+^ entry into mitochondria and nuclei [96]. In mitochondria Ca^2+^ cause the activation of mitochondrial metabolism that can switch from a physiological beneficial process to a cell death signal whereas in nuclei Ca^2+^ modulate gene transcription and nucleases that control cell death. Moreover Stout et. al. have experimentally shown that increased levels of Ca^2+^ in the cytoplasm are not necessarily toxic if the Ca^2+^ uptake by mitochondria is inhibited. [101]. Therefore this indicates that mitochondria are important targets for switching normal Ca^2+^ signaling to signals for cell death during severe oxidative stress.

## 6. Antioxidant Defense System

Several defense mechanisms have been developed to protect against exposure to different free radicals [102], such as physical, repair, and preventive mechanisms. The antioxidant defense mechanism comprises two components: (1) enzymatic components including catalases, SOD, and glutathione peroxidase and (2) nonenzymatic components including vitamin C, vitamin E, carotenoids, glutathione, and flavonoids, among others. Various reviews and research papers have indicated the role and mechanism of both enzymatic and nonenzymatic components in protecting against oxidative stress [103–116].

Consider the case of GSH, which acts as a redox buffer inside a cell [117]. GSH is found in almost every cell compartment, including the cytosol. GSSG represents the oxidized form of GSH inside a cell. Therefore, measuring the ratio of GSH to GSSG can provide a good indication of the oxidative stress [118, 119]. The GSH inside the nucleus helps maintain the redox of sulfhydryl proteins, which are important for repair and expression. When a cell is treated with GSH, it is readily taken by the mitochondria against the concentration gradient. GSH also plays an important role [117] in activating vitamin C and vitamin E and transporting amino acids through the plasma membrane. It scavenges singlet oxygen and hydroxyl radicals, detoxifies hydrogen, and lipid peroxide and is a cofactor in several detoxifying enzymes.

## 7. Oxidative Stress in Human Immunodeficiency Virus (HIV)

Oxidative stress has been found to occur in various viral infections [120–123] that may enhance viral replication. In an* in vitro *condition, oxidative stress has been found to enhance HIV replication [124–126]. The nuclear transcription factor NF-*κ*B, which is necessary for viral replication, is activated when oxidative stress is present [124, 127]. The other role of NF-*κ*B is to activate many of the immune system's inflammatory cytokines [128, 129]. Many antioxidants have been examined to determine their antiviral activities. However, due to unknown reasons, they have been shown to have varying effects in different cell culture systems and have shown no improvement even when examined* in vitro* at higher concentrations. HIV-infected and AIDS patients have exhibited elevated serum levels of hydroperoxides and malondialdehyde, which are the byproducts of lipid peroxidation [130–133], in addition to membrane damage. They have also exhibited an increase in resting oxygen consumption, as free-radical formation is linked to oxygen metabolism [133]. This information is supported by the production of ROS in the neutrophiles of HIV-infected patients [134], whose antioxidant defense systems undergo dramatic changes. Children suffering from HIV infection have exhibited decreased SOD levels and activity [135]. Antioxidant enzyme catalase activity increases as AIDS progresses in HIV-infected patients [136]. The level of glutathione peroxidase in RBCs and plasma also decreases. This clearly shows that the body antioxidant system becomes weaker as HIV progresses. The imbalances inside and outside the cell influence the cell to undergo a programmed cell death. The weakening of the body's antioxidant components such as catalase and glutathione leads to an excess storage of H_2_O_2_, which further increases the hydroxyl radicals and lipid peroxide that signal the cell to undergo a programmed cell death [137]. In* in vitro* conditions, the additions of H_2_O_2_ and antioxidants result in a respective increase and decrease in apoptosis in the cell culture system. AIDS, which is characterized by a decrease in the CD4 lymphocytes, is currently believed to be the main culprit of this apoptosis [138, 139]. The imbalance in the ROS seems to contribute to the progression of AIDS in different ways, including the apoptosis of CD4 cells and the functioning of other immune system components [140].

### 7.1. Envelop Glycoprotein “Gp120” of Human Immunodeficiency Virus-1 in ROS Production

HIV-1 uses glycoprotein (gp120) to enter host cells (T-cells and monocytes). Infected monocytes can cross the blood-brain barrier (BBB) and finally replicate in astrocytes and microglia [141, 142]. Recent work has shown that HIV-1 induces ROS production (oxidative stress) in astrocytes and microglia [143, 144] and that gp120 can directly induce apoptosis in neurons [145]. It has also been shown recently that the involvement of P450 (CYP) in neurotoxicity may be due to the generation of ROS or other reactive metabolites [146]. Furthermore, gp120 along with the drug methamphetamine (MA) involves CYP and NOX pathways in apoptotic cell death. Both gp120 and MA have been found to cause oxidative stress due to the production of ROS concentrations in a time-dependent manner [147]. The ROS-mediated BBB damage in the HIV-1 infection has been shown to cause a loss of cell tight junction proteins and lipid per oxidation [148, 149]. MA and gp120 together cause a loss of tight junction proteins in BBB and make it leaky, facilitating the entry of infected monocytes [149]. MA increases oxidative stress through dopaminergic and glutamatergic mechanisms [150], and gp120 increases oxidative stress through glutathione and lipid per oxidation [144, 151]. A combination of cocaine and gp120 results in an excess production of ROS that in turn activate caspase-3 and NF-*κ*B to force the astrocytes to undergo apoptosis [152]. In addition to considering the role of ROS in different diseases [153], recent reports have shown that oxidative stress is involved in the pathology of HIV-associated neurocognitive disorders [154]. The role that CYPs play in different tissues/organs including the brain [155] has also been confirmed. Astrocytes have been shown to express many CYPs at variable levels, and the roles of CYP2E1 and CYP2A6 in alcohol- and nicotine-mediated oxidative stress have been demonstrated [147, 156, 157]. MA has been shown to cause increased expressions of CYP2A6, 2B6, and 2D6; gp120 has been shown to cause increased expressions of CYP2E1, 2B6, and 2D6 [158]. These overall additive increases suggest that CYP may be involved in oxidative stress. The interaction between CYPs and NADPH is tightly regulated by NOX enzymes [159], which are currently being used as therapeutics in various CNS disorders such as Alzheimer's disease and strokes [159, 160]. Studies have shown that NOX 2 and NOX 4 increase oxidative stress in astrocytes [161, 162]. Others have shown that when NOX2 and NOX4 expression is blocked in astrocytes, the level of oxidative stress decreases, indicating that NOX could be used as a therapeutic agent in the treatment of neuro-AIDS. Such studies of oxidative stress in astrocytes caused by MA/gp120 have examined the use of antioxidants in HIV-1 pathogenesis and considered the potential of CYP pathways to be a target of new drugs.

## 8. ROS in the Hepatitis C Virus (HCV)

HCV infection cases have been reported around the world and are increasing at an alarming rate, especially in developing nations. Reports have shown that 3% of the world's population is infected with HCV [163]. Although acute hepatitis caused by HCV is naturally cleared in 20–30% of patients [164], 70–80% of cases involve chronic hepatitis. No effective vaccine was available until recently, and the current treatment is not very effective [165, 166]. Reports have shown that HCV gene expression in the host cell increases the level of ROS through the mediation of calcium signaling [167]. This release of calcium from the ER results in ER stress. The released calcium is taken by the mitochondria, resulting in increased ROS production and oxidative stress. Oxidative stress is the main contributor to a number of diseases such as cancer [168], diabetes, and even viral infections [169]. The livers of patients with HCV infections show elevated levels of ROS and decreased antioxidant levels [170]. It has been reported that the two core proteins of HCV, NS3 and NS5A, are responsible for oxidative stress in culture cells [171]. However, the host cell Cox-2 gene, which is the main regulator of prostaglandins, is activated by the excess ROS produced [172]. This activation involves NF-*κ*B, which is present in cells in an inactive form but becomes activated and migrates to the nucleus in HCV-infected cells due to ER stress and ROS [173]. NF-*κ*B controls the expression of the genes responsible for apoptosis and inflammation. This elevated level of ROS activates another transcriptional factor (STAT-3) that is responsible for cell proliferation, survival, and ontogenesis [174]. This coactivation of both NF-*κ*B and STAT-3 as a result of the oxidative stress created by excessive ROS in HCV-infected cells has an equal role in both acute and chronic liver diseases [169, 173, 175].

### 8.1. HCV Genome in ROS Production

The liver plays an important role in the detoxification and metabolism of harmful substances and is the main target of HCV. HCV replicates in cytoplasm, causing hepatitis cirrhosis and hepatocellular carcinoma [176, 177]. ROS-induced viral genome heterogeneity has been considered in terms of viral escape from the immune system [178]. The core nucleocapsid protein of HCV is responsible for increasing oxidative stress in the liver [179]. Although this core protein is considered the main contributor of oxidative stress [180, 181], other proteins such as NS3 and NS5A are also involved in generating oxidative stress [182–184]. Recent studies have shown that many other proteins such as E1 [180], E2 [185, 186], and NS4B [182, 187] are also involved in generating oxidative stress. The nonstructural protein NS5A is a membrane integral protein that is important not only for viral replication but also for apoptosis and immune responses such as interferon resistance [188] and changes in calcium levels. NS5A and NS3 increase the calcium uptake and cause glutathione oxidation in mitochondria, thereby increasing the ROS production [189–191]. The mitochondria thereby activate and translocate the transcriptional factors NF-*κ*B and STAT3 to the nucleus, leading to oxidative stress. The NS5A activation of NF-*κ*B and STAT3 is opposed by antioxidants [192, 193]. NS4B also translocates NF-*κ*B to the nucleus in a PTK-mediated pathway. ROS and NO^*∙*^ not only cause oxidative damage but also affect the DNA repair machinery [194–196] that leads to cell apoptosis. ROS are believed to be the main culprits of liver inflammation in HCV infections [197, 198].

## 9. ROS in Influenza Virus

The influenza virus induces the production of ROS in host cells that can damage the virus genome [199]. ROS enhance the pathogenesis ability of infections such as influenza [200, 201]. One study of mice infected with the influenza virus showed that although the spread of infection remained confined to the airways and lungs [201], systematic effects such as weight loss and a decrease in body temperature were clearly visible. The mice used in the experiment died after 5 or 6 days. The cells taken from the dead mice showed elevated levels of O_2_^−^ and xanthine oxidase (an enzyme synthesizing O_2_^−^), indicating enhanced ROS production [202]. Furthermore, analysis of the antioxidant content revealed an overall decrease in the concentration of antioxidants during infection. The study suggested that influenza infection is associated with oxidative stress. In another study, influenza intravenously injected into mice with pyran-copolymer-conjugated SOD was found to protect the mice from the effects of influenza. This observation was not immediately apparent because pyran copolymers are well-known antiviral agents [203]. The localized effects of the influenza virus make it difficult to detect the redox content of tissues because the analysis methods are based on whole-tissue homogenates. The infected mice released cytokines and lipid mediators that could have caused the systematic symptoms [204]. To determine the cause of the systematic symptoms, the mice injected with the influenza virus were given cytokine injections (mostly interferon) and showed symptoms resembling influenza [205]. ROS are known for their antiviral activity [206] and can also increase the titer of the influenza virus. Influnza virus carrying glycoprotein on its surface is know as hemagglutinin which is responsible for binding the virus to cells with sialic acid on their membranes, like cells in the upper respiratory tract or erythrocytes [207]. The hemagglutinin protein is synthesized in an inactive form (HOA) and activated by specific proteases into HA1 and HA2. The cleavage of HOA into HA1 and HA2 is an important determinant of influenza virulence [207, 208]. If the influenza virus released from the cell contains inactive HOA, it may still be activated by some of the proteases present in the pulmonary surfactants [209]. However, these antiproteases can be inactivated by the ROS, converting a noninfectious influenza virus into an infectious one. Further studies have shown that an oxidant-treated antiprotease is unable to prevent trypsin from converting HOA to HA1 and HA2, resulting in a 10,000-fold increase in virus infection [207]. However, how the influenza virus induces apoptosis is still not clearly understood.

ROS production enhances the molecular pathogenesis of the influenza virus infection. Previous research has proved that although ROS are involved in damaging lung parenchyma, that damage can be repaired by taking an appropriate dose of antioxidants [209, 210]. ROS are important in the overall normal development of whole organisms [211, 212], are important components of adaptive immune responses, and are involved in the normal function of many transcription factors. The production of ROS (superoxide's) is an important defense against microbial infections. However, the excess production of superoxide's in the influenza A virus infection is detrimental. The downregulation of the superoxide achieved by targeting specific enzymes such as NADPH oxidase-2 markedly alleviates lung injuries caused by the influenza virus and viral replication, irrespective of the infected viral strain [213]. One study showed that influenza infection leads to the thymus-specific elevation of the mitochondrial superoxide, which interferes with the normal functioning of T-cell lymphocyte damage in influenza A virus infections [214]. A further knockdown of SOD2 indicates that T-cells begin the apoptosis process and take on many developmental defects, resulting in overall weakening of the adaptive immune system and an increased susceptibility to the influenza A virus (H1N1). Keeping the use of ROS as specific targets in mind, ROS inhibitors and other therapeutic agents may prove useful in controlling such a disease [215].

## 10. ROS Production in Fish Virus Infections

### 10.1. Betanodavirus (Mitochondria as the ROS Production Houses in Infected Cells)

The Betanodavirus is an RNA virus belonging to the Nodaviridae family, which mostly infects fish. The virus causes virus nervous necrosis (VNN) disease, which is characterized by the necrosis of the central nervous system, including the brain and retina. The common symptoms shown by infected fish are abnormal swimming behavior, darkening of the skin, and weight loss [216]. The viral capsid protein [217] is involved in the postapoptotic necrotic cell death via a cytochrome C release-dependent pathway [218]. Research has shown that the majority of RNA viruses, DNA viruses, and retroviruses cause ROS-mediated cell death. The Betanodavirus genome encodes protein alpha and B2, both of which are death inducers. Protein alpha causes mitochondria-mediated cell death involving caspase-3 [219], and B2 does the same via a Bax-mediated pathway [220]. Another protein, B1, acts as an antinecrotic death gene [221]. Our research shows that the production of ROS partly causes mitochondria-mediated cell death in RGNNV-infected cells [94]. This supports previous research related to the involvement of oxidative stress in cell deaths caused during RNA virus infection. It opens doors for the development of new drugs by making the enzymes or other key factors involved in ROS production the main targets. Mitochondria are the main production houses of ROS during RGNNV infection, which ultimately leads to mitochondria-mediated cell death [94, 222]. Mitochondria complexes I and II of the electron transport chains are the major sites of ROS production [222, 223]. The inhibition of complexes I (rotenone) and II (antimycin) and the oxidation of either complex both lead to increased ROS production [222, 224–226]. However, ROS are also important in the activation of the body's antioxidant enzymes such as SOD and glutathione peroxidase [127, 227]. RGNNV-infected cells were found to produce ROS at 24 h after infection, with a gradual regulation of catalase and Nrf2 transcription factors [228] and autophagy (unpublished data). However, it remains unclear whether Nrf2 upregulates ROS production. We used antioxidants such as NAC and DPI and overexpressed zfcatalase to further explore our hypothesis and found a decrease in RGNNV-induced ROS production and an increase in cell viability. The cell death mechanism influenced by the novel antinecrotic cell death protein B1 remains unknown. To determine which cell death mechanism is influenced by B1, we examined how the Betanodavirus nonstructural protein B1 regulates oxidative stress and p53 expression in fish cell lines [94].

## 11. Conclusion and Future Perspective

Viral infections are becoming more common daily around the world. People living in the poorest countries represent the most infected population due to their unhygienic food conditions, illiteracy, and lack of basic health care. Identifying the main culprit of new epidemics is the most important factor in controlling the outbreak of disease. Many host mechanisms have been shown or are suspected to contribute to the pathogenesis of viral infections, such as ROS and cytokines. Current studies of ROS are based on the lethal effects of ROS in various diseases such as cancer, HIV, hepatitis, and diabetes. The discovery of* “respiratory bursts”* revealed that ROS are only produced by phagocyte cells to protect against microbial invasions and are thus considered toxic molecules. However, recent cellular ROS studies have shown that ROS are produced in all types of cells and serve as important messengers in cell signaling and various signal transduction pathways. The ROS produced inside cells are maintained by complex intracellular regulatory systems. Cells respond to the ROS they produce according to different parameters such as intensity, duration, and amount. Therefore, the combination of several mechanisms described in this review could be exploited to find new solutions for combating oxidative stress in different viral infections. However, the role of oxidants in viral diseases is more complex because it includes metabolic regulations for both host metabolisms and viral replication. A number of different additional host mechanisms have been shown or are suspected to contribute to the pathogenesis of viral infections, including excessive cytokine, lipid peroxidation, lipid mediator release, and compliment activation [15, 229]. Therefore, more deep and detailed research is needed to interfere with the activation or stop the undesired effects of these pathways. The limitations of interfering with such viral disease mechanisms are similar to those involved in interfering with oxidant generation, as these pathways are associated with normal host physiology and pathology. It is clear that any useful approach to solving this problem will require a variety of drugs rather than two or three drugs according to modern pharmacotherapy. Clarifying the role of oxidation stress in apoptosis could lead to a discovery of novel therapeutic strategies and pathogenetic insights into different viral diseases, particularly given that ROS-mediated mechanisms are responsible for apoptosis during viral infections. ROS were initially detected based on simple absorbance measurements. Due to the discovery that ROS act as intracellular messengers and regulators, the absorbance-based detection was replaced by fluorescence- and luminescence-based forms of detection that are more specific and accurate and less time consuming. The present-day cellular ROS research faces great difficulty in detecting ROS due to the lack of reporter agents against these molecules. Because ROS molecules are highly reactive with most other molecules, designing reporter agents has been difficult. Some of the challenges involved in researching cellular ROS in different diseases include the following.

- Although much of the available evidence supports the involvement of ROS in lung injuries caused by the influenza A virus, the molecular mechanism and enzymes for ROS production remain unclear. Thus, knowing the enzymes and other key factors could be the main aim in designing new drugs against the influenza infection.
- Studying the oxidative stress in HIV-infected patients has opened new doors for cellular ROS researchers to use antioxidants as novel drugs to decrease HIV-1 pathogenesis in humans.
- The current cellular ROS research does not provide enough proof to show the exact relationships among mitochondria functions, ROS production, ROS damage, and the development of clinical phenotypes.
- Research has shown that ROS are the main regulatory factors in a number of molecular pathways, especially those linked to the development and spreading of tumors. Thus, studying ROS as the main therapeutic targets could be made a focus for controlling disease.
- The role of ROS and mitochondria in neurodegenerative diseases and aging is also a matter of interest, as the oxidative stress generated by an ROS imbalance can be a consequence rather than a cause of the disease process.
- Studying the detailed mechanisms of N0^*∙*^/redox-mediated signaling will help in the development of novel therapeutic approaches to addressing heart failure.
- It remains unknown how much of the mitochondrial damage in Parkinson's disease cases is of genetic origin and how much is caused by the H_2_O_2_ generated in the dopamine produced by neurons.

As discussed previously, much of the available evidence indicates that free radicals play a complex role in different viral diseases, beginning with their influence on the host cell's metabolism and viral replication and extending to their desired inactivation effects on viruses and less-desired toxic effects on host tissues. The use of antioxidants in viral disease therapy could therefore be applied at many levels and replace the old symptomatic therapy, which would not alter the viral replication. The new therapy should also target additional mechanisms that contribute to the symptoms and pathology of viral diseases such as cytokines, lipid peroxidation, and NO^*∙*^. Most virus-induced ROS generation is linked to the activation of different signaling molecules and transcription factors such as NF-*κ*B, STAT (STAT1, STAT3), and JAK (JAK2). However, the intracellular signaling events that lead the viral-induced gene expression are mostly unknown. Some ROS researchers have described ROS as secondary messengers that influence a number of different molecular processes, including the apoptotic, antiapoptotic, and proapoptotic expression of a number of genes. The physiological role played by ROS is important because viruses depend on the biosynthetic mechanisms of their host cells as intracellular parasites. The activation of ROS production in viral infections in the absence of antiviral antibodies could play a role in the generation of symptoms and pathologies such as the induction of fever in the influenza virus and could also lead to internal organ hemorrhages. Therefore, the main challenge for present-day molecular virologists is to understand the pathophysiological functions of ROS, which would provide deep understanding of the many aspects of viral infectious diseases. The effect of ROS on the host's immune response is another important factor of viral pathogenesis and mutation. The toxicity and reactivity of ROS, which are produced in excess amounts by the overreactions of immune responses against the organs or tissues in which viruses replicate, may explain the tissue injury mechanisms observed in the different viral diseases involving immunological interactions. Understanding of the host pathogen interactions at the molecular level requires the characterization of host-derived small radical molecules, which appear to play an important role in the pathogenesis of viral infection. An energizing concept related to free radicals would contribute to the insights into the molecular mechanisms of pathological events that occur as a result of the interaction between viruses and their hosts. Therefore, more deep and detailed research must be conducted to better understand the molecular mechanism and specific apoptotic pathways involved in ROS-mediated cell death. The growing interest shown by cellular ROS researchers should provide answers for many of these unsolved questions.
